# The Acute Phase of *Trypanosoma cruzi* Infection Is Attenuated in 5-Lipoxygenase-Deficient Mice

**DOI:** 10.1155/2014/893634

**Published:** 2014-08-03

**Authors:** Adriana M. C. Canavaci, Carlos A. Sorgi, Vicente P. Martins, Fabiana R. Morais, Érika V. G. de Sousa, Bruno C. Trindade, Fernando Q. Cunha, Marcos A. Rossi, David M. Aronoff, Lúcia H. Faccioli, Auro Nomizo

**Affiliations:** ^1^Departamento de Análises Clínicas, Toxicológicas e Bromatológicas, Faculdade de Ciências Farmacêuticas de Ribeirão Preto, Universidade de São Paulo, Avenida do Café, s/n, 14040-903 Ribeirão Preto, SP, Brazil; ^2^Departamento de Biologia Celular, Instituto de Ciências Biológicas, Universidade de Brasília, Campus Darcy Ribeiro, 70910-900 Brasília, DF, Brazil; ^3^Departamento de Farmacologia, Faculdade de Medicina de Ribeirão Preto, Universidade de São Paulo, Avenida Bandeirantes, No. 3900, 14049-900 Ribeirão Preto, SP, Brazil; ^4^Departamento de Patologia, Faculdade de Medicina de Ribeirão Preto, Universidade de São Paulo, Avenida Bandeirantes, No. 3900, 14049-900 Ribeirão Preto, SP, Brazil; ^5^Division of Infectious Diseases, Department of Medicine, Vanderbilt University Medical Center, Nashville, TN 37232, USA

## Abstract

In the present work we examine the contribution of 5-lipoxygenase- (5-LO-) derived lipid mediators to immune responses during the acute phase of *Trypanosoma cruzi* infection in 5-LO gene knockout (5-LO^−/−^) mice and wild-type (WT) mice. Compared with WT mice, the 5-LO^−/−^ mice developed less parasitemia/tissue parasitism, less inflammatory cell infiltrates, and a lower mortality. This resistance of 5-LO^−/−^ mice correlated with several differences in the immune response to infection, including reduced PGE_2_ synthesis; sustained capacity of splenocytes to produce high levels of interleukin (IL)-12 early in the infection; enhanced splenocyte production of IL-1*β*, IL-6, and IFN-*γ*; rapid T-cell polarization to secrete high quantities of IFN-*γ* and low quantities of IL-10; and greater numbers of CD8^+^CD44^high^CD62L^low^ memory effector T cells at the end of the acute phase of infection. The high mortality in WT mice was associated with increased production of LTB_4_/LTC_4_, T cell bias to produce IFN-*γ*, high levels of serum nitrite, and marked protein extravasation into the peritoneal cavity, although survival was improved by treatment with a cys-LT receptor 1 antagonist. These data also provide evidence that 5-LO-derived mediators negatively affect host survival during the acute phase of *T. cruzi* infection.

## 1. Introduction

Infection with* Trypanosoma cruzi (T. cruzi)*, an obligate intracellular protozoan parasite, causes American trypanosomiasis or Chagas disease, a zoonosis endemic to Latin America. Approximately 60 million people live in areas with vector-borne transmission risk and the disease causes an estimated 14,000 deaths per year [1]. After entering the host,* T. cruzi* invades a variety of cell types, such as macrophages, heart muscle cells, skeletal muscle cells, and neurons, replicating within the cytoplasm [2]. The acute phase of the disease is characterized by a marked increase in parasite replication and migration to the blood, potentially leading to systemic infection. However, immunocompetent hosts are able to generate innate inflammatory and specific immune responses to acute secondary infection, thereby controlling the parasite burden [3]. These responses are primarily dependent on cytokine/chemokine mediated activation of infected phagocytes and/or tissue cells which leads to intracellular killing [4], although complete elimination of the parasite is rarely achieved. Parasite persistence in tissues is followed by an asymptomatic or indeterminate phase, and chronic chagasic immunopathology develops in approximately 25% of cases [5].

The factors governing immunological resistance to acute trypanosomiasis are not fully understood. Host genetic background and parasite strain differences might be relevant [6]. Early, partial control of parasites within infected tissue is achieved by local production of type 1 IFNs [7], IL-1*β* [8], and *β*-chemokines [9]. Therefore, effective parasite control likely requires the participation of both innate and adaptive immune cells including macrophages, dendritic cells, and NK cells that secrete proinflammatory cytokines (e.g., IL-12 or IFN-*γ*) [10] and naive T cells for the generation of parasite-specific CD4^+^ and CD8^+^ effector T cells [11], which produce Th1 cytokines such as IFN-*γ* and, in lesser quantities, Th2 cytokines such as IL-4 and IL-10 [12, 13].

Although immune functions have been assigned to a number of polypeptide mediators (cytokines and chemokines) in host defense against* T. cruzi*, little attention has been paid to the role of lipid mediators. These lipid molecules are mainly eicosanoids that are generated through the effects of cyclooxygenases (COX) or 5-lipoxygenase (5-LO) and play a variety of roles in regulating host innate and adaptive immune responses [14]. The 5-LO pathway leads to the formation of two biologically relevant classes of leukotrienes (LTs): non-cysteinyl LTs such as LTB_4_; and cysteinyl-LTs (cys-LTs) such as LTC_4_, LTD_4_, and LTE_4_ [15]; and the activity of 5-LO seems to be a common step in LXA_4_ synthesis [16]. LTs have been established to play protective roles during infection with many microbial pathogens, including* Salmonella typhimurium, Pseudomonas aeruginosa* [17],* Klebsiella pneumoniae* [18], vesicular stomatitis virus encephalitis [19], and* Histoplasma capsulatum* [20]. However, in other settings 5-LO products have been shown to play contradictory roles, for example, in* Mycobacterium tuberculosis* infection models [21, 22]. In addition, in a cecal ligation and puncture model of peritonitis, LTs exhibited beneficial effects on local immunity but exhibited deleterious effects on hemodynamic responses [23]. Immunoregulatory lipids, such as the arachidonic acid-derived eicosanoids, are increasingly implicated in the pathogenesis of parasitic infections [24, 25]. The 5-LO pathway products have also been implicated in modulating the pathogenesis of several parasitic infections and the results have also been contradictory.* In vitro*, LTB_4_ and LTC_4_ potentiate macrophages to kill* T. cruzi *[26, 27] and* Leishmania amazonensis* [28]. However, these mediators have been implicated in conferring susceptibility to* Schistosoma mansoni* [29],* Strongyloides venezuelensis* [30], and cerebral malaria [31], thereby suggesting that LTs play conflicting roles during parasite infection.

The immunoregulatory effects of 5-LO pathway eicosanoids are complex and context dependent. While their net effects are beneficial to host defense against some microbial pathogens, this is not necessarily true for all infections. In light of the importance in regulating immune responses to parasitic infections, and the contrasting roles exhibited by LTs in several infection models, we asked whether the 5-LO pathway activity could modulate the* T. cruzi* infection. To address this issue, here we studied specifically the acute phase of* T. cruzi* infection in 5-LO^−/−^ mice.

## 2. Materials and Methods

### 2.1. Animals

Male mice (18–20 g) were used; the 5-LO^−/−^ (129-*Alox5* ^*tm1Fun*^ */J*) and strain-matched WT mice (129-SF2/J) were purchased from Jackson Laboratories (Bar Harbor, ME). The animal colony was bred and maintained under specific pathogen-free conditions at the Faculdade de Ciências Farmacêuticas de Ribeirão Preto (Universidade de São Paulo, Brazil). This study was approved and carried out in strict accordance with the guidelines of the Animal Care Committee of the Universidade de São Paulo and Biosafety Committees (Process nos. 05.1.592.53.2 and CQB-0019/97). All euthanasia was performed under CO_2_/O_2_ excess atmosphere, and all efforts were made to minimize suffering.

### 2.2. Parasite Infection and Pharmacological Treatment

Mice were infected intraperitoneally with 200 units of the blood form of* T. cruzi* (Colombian strain) in 0.2 mL of 0.15 M PBS. Control mice received the same volume of sterile PBS. Parasites were counted in 5 *μ*L of blood as previously described [32]. WT mice were previously subjected to* T. cruzi* infection [33]. In some experiments, the infected WT mice were treated with a cys-LT receptor 1 antagonist, montelukast (10 mg/kg, Singulair; Merck Sharp & Dohme, Campinas, Brazil) or its vehicle, carboxymethylcellulose (0.5% w/v), administered orally by gavage (300 *μ*L/animal) on postinoculation days 14–32, starting on day 14 and given every 2 days.* T. cruzi* soluble antigens were obtained from trypomastigote forms (Colombian strain) and used for* in vitro* experiments [32]. Briefly, trypomastigotes were washed twice in cold PBS, subjected to six freeze-thaw cycles, and centrifuged (9000 ×g, 10 min, 4°C). The supernatant was filtered through a 0.22 *μ*m pore size membrane filter, and the protein concentration was measured using a colorimetric assay (Pierce, Rockford, IL).

### 2.3. Histology and Quantitative Tissue Parasite Nest Determination

Histology and tissue parasite counts were performed as described elsewhere [11]. Briefly, tissue samples were fixed in 4% buffered formalin and processed for conventional paraffin embedding on day 16 after infection. Sections (8 *μ*m) were deparaffinized and stained with hematoxylin and eosin. Intact parasite nests were evaluated in blinded samples by counting the number of parasites nests in 100 microscopic fields/sample of nonconsecutive sections.

### 2.4. Eicosanoid Levels in Peritoneal Cell Supernatants

Peritoneal cells were collected by intraperitoneal injection of 4 mL of cold PBS from uninfected controls, infected WT, and 5-LO^−/−^ mice at various time points of infection. Cell concentration was adjusted to 10^6^ cells mL^−1^ in Hank's buffered salt solution (HBSS; Sigma, St. Louis, MO) with Ca^+2^ and Mg^+2^. Cells were stimulated with 0.5 *μ*M of the calcium ionophore A23187 (Sigma, Saint Louis, EUA) for 15 min at 37°C in a humidified atmosphere of 5% CO_2_. The supernatants were harvested and PGE_2_, LTB_4_, and LTC_4_ levels were determined by specific EIA kit, following the manufacturer's instructions (GE Healthcare, Little Chalfont, UK).

### 2.5. Spleen Cell Culture

Mice from experimental groups were euthanized on various days after inoculation. Single-cell suspensions were prepared by passing each spleen through a 70 *μ*m cell strainer (Falcon, Sollentuna, Sweden). The splenocytes were washed 3 times with HBSS, counted with a hemocytometer, assessed for viability, and suspended in RPMI 1640 medium supplemented with 10% FCS, penicillin (100 U/mL), streptomycin (100 *μ*g/mL), and gentamicin (50 *μ*g/mL) (Gibco-Invitrogen, Carlsbad, CA) or HBSS supplemented with 5% FCS. The cell concentration was adjusted to 10^7^ cells/mL and cultured in 24-well plates (Nalge Nunc, Rochester, NY) in 1 mL of supplemented RPMI medium, with 5 *μ*g of anti-CD3*ε* or with 10–50 *μ*g of soluble* T. cruzi* antigens at 37°C in an atmosphere of 5% CO_2 _for 24–48 h. Supernatants were collected and stored at −70°C for further use.

### 2.6. Metabolic Assays

Splenocytes (4 × 10^5^ cell/well) from different experimental groups were cultured in quintuplicate in flat 96-well microplates (Nalge Nunc, Rochester, NY) with supplemented RPMI medium. Cells were cultured alone or with anti-CD3*ε* IgG (1 *μ*g/mL; BD Pharmingen, San Diego, CA) at 37°C in a humidified atmosphere of 5% CO_2_. After 60 h, 10 *μ*L (5 mg/mL) of MTT (Sigma, Saint Louis, EUA) was added to each well, and cells were incubated for an additional 4 h, followed by the addition of 50 *μ*L of 20% SDS in PBS and stored in the dark overnight. Absorbance was measured at 570 nm using an automated microplate reader (*μ*QUANT; BioTek Instruments, Winooski, VT).

### 2.7. Flow Cytometry

Spleen cells were isolated as described above and placed in ice-cold PBS supplemented with 5% FCS and 0.1% sodium azide. Staining was performed as previously described [11]. The following fluorochrome-conjugated monoclonal antibodies were used: anti-CD4 [H129.19]; anti-CD8 [53-6.7]; anti-CD19 [MB19-1]; anti-CD25 [7D4]; anti-CD44 [IM7]; anti-CD69 [H1.2F3]; anti-Gr-1/Ly6C/Ly6G [RB6-8C5]; anti-CD45RB [16A]; anti-CD62L [MEL-14]; anti-CD11b [M1/70] (BD Pharmingen, San Diego, CA); and anti-CD11c [HL3]—(Serotech, Raleigh, NC) anti-F4/80 [CI:A3-1] and anti-GITR [DTA-1] (eBioscience, San Diego, CA). After staining, the cells were fixed with 1% paraformaldehyde in PBS and analyzed using a FACSCanto (BD Biosciences, San Jose, CA), 50,000 events/sample recorded. Data were processed using FlowJo software (FlowJo LLC, Ashland, OR). Cell numbers were calculated using the percentage obtained by FACS analysis and the total numbers of leukocytes counted in a hemocytometer.

### 2.8. *T. cruzi*-Specific Antibodies

Specific IgG, IgG1, and IgG2a were determined in mouse sera by ELISA as previously described [32]. The individual titers were considered the highest serum dilutions that presented OD_492_ > 0.1.

### 2.9. Protein Extravasation

Protein extravasation was assessed as previously described [23]. Control mice or infected mice were i.v. injected with Evans blue dye (50 mg/kg in a volume of 0.1 mL; Sigma, Saint Louis, EUA). After 1 h, mice were euthanized by CO_2_ inhalation, and the peritoneal exudates were recovered by injecting 2 mL of PBS. The peritoneal exudates were centrifuged for 10 min at 200 ×g, and the supernatant was saved for colorimetric determinations. The OD was determined at 630 nm in the automated microplate reader.

### 2.10. *In Vitro* Macrophage Infection

Peritoneal cells from WT and 5-LO^−/−^ mice were collected, washed twice, and counted and the cell concentration was adjusted to 10^6^cells/mL in supplemented RPMI medium. Cells were attached on 13 mm-diameter glass coverslips placed to 24-well plates (Nalge Nunc, Rochester, NY), for 90 min at 37°C in an atmosphere of 5% CO_2_. The nonadherent cells were removed by washings in warm supplemented RPMI medium. Peritoneal macrophages (PM) isolated by this procedure were >90% pure as measured by staining for F4/80^+^ (data not shown). The PMs were stimulated for 6 h with 5 ng/mL of IFN-*γ* (BD Pharmingen, San Diego, CA) plus 0.1 *μ*g/mL of LPS from* Escherichia coli* (Sigma, Saint Louis, EUA) and infected at a parasite-to-macrophage ratio of 5 : 1/well. After 2 h, the glass coverslips were washed five times in PBS to remove free parasites, fixed in absolute methanol, stained with Panoptic stain (Laborclin, Pinhais, Brazil), dried, mounted on glass slides, and examined microscopically for association (parasite adhered to macrophages plus internalized parasites) as previously described [26]. For killing assay, noninternalized parasites of infected macrophages wells were removed 24 h later by three gentle washes with warm supplemented RPMI medium. Fresh supplemented RPMI medium was added to each well, and infected macrophages were cultured at 37°C, in an atmosphere of 5% CO_2_ for up to 10 days, 50% of the medium being removed and replaced with the same volume of supplemented RPMI medium every 48 h. After 7–11 days after infection, culture supernatants were collected daily to count the number of motile trypomastigotes/well.

### 2.11. Nitrite/Nitrate Concentration

Tail-vein blood samples were obtained at day 22 after inoculation. Nitrate in serum samples was converted to nitrite by nitrate reductase, and serum levels of nitrate/nitrite (Nitric oxide end-products or metabolites) were measured by absorbance using the Griess Reaction (Calbiochem, La Jolla, CA) [34]. The OD was determined at 540 nm in the *μ*QUANT automated microplate reader. The nitriteconcentration was determined by reference to a standard (1–100 *μ*M) sodium nitrite curve.

### 2.12. Cytokine ELISA

Levels of IL-1*β*, IL-2, IL-6, IL-10, IL-12, TNF-*α*, and IFN-*γ* were quantified by ELISA according to the manufacturer's instructions (BD Pharmingen, San Diego, CA) in the splenocytes culture supernatant. The lower limits of detection for those cytokines were 9.4 pg/mL.

### 2.13. Statistical Analysis

The results are presented as means ± SD, with the exception of those for parasitemia, shown as means ± SEM. The tests that were used to evaluate differences among groups are mentioned in the figure legends. Values of *P* < 0.05 were considered significant.

## 3. Results

### 3.1. Lipid Mediator Production by Infected Peritoneal Cells

**Figure 1:**
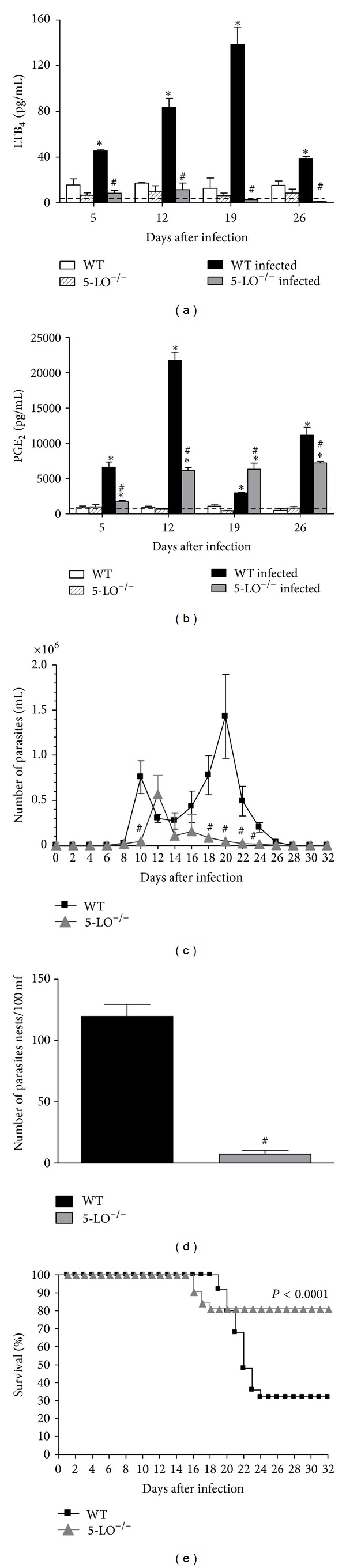
Lipid mediator production, parasitemia, tissue parasitism, and survival rate of WT and 5-LO^−/−^ mice infected with* T. cruzi*: ((a) and (b)) LTB_4_ and PGE_2_: peritoneal cells were collected from control, infected WT, and infected 5-LO^−/−^ mice (*n* = 10/group) and stimulated with calcium ionophore. *P < 0.01 versus uninfected group; ^#^ *P* < 0.01 versus WT infected mice. (c) Parasitemia (*n* = 10 mice/group). ^#^ *P* < 0.001 versus infected WT mice. (d) Parasite nests in heart tissue on postinoculation day 16. *P < 0.001 versus infected WT mice. (e) Survival: WT mice (squares) and 5-LO^−/−^ mice (triangles), *n* = 10 animals/group. Wilcoxon signed-rank test (level of significance, *P* < 0.001). Data are representative of three independent experiments.

Peritoneal cells from* T. cruzi*-infected mice (WT) released significantly more LTB_4_ and PGE_2_ upon calcium ionophore stimulation than did cells obtained from uninfected mice (Figures 1(a) and 1(b)). The potential of cells to produce LTB_4_ was elevated at an early time point after inoculation (day 5) and increased gradually throughout the infection, peaking on day 19. This potential then decreased drastically toward the late phase of acute infection (day 26; Figure 1(a)).

The potential of peritoneal cells from* T. cruzi*-infected mice to produce PGE_2_ was also increased (Figure 1(b)). Peritoneal cells from infected WT mice released PGE_2_ at early time points (day 5) and increased markedly on subsequent days, peaking on day 12. PGE_2_ levels decreased drastically by day 19 and increased again in the late phase of the acute infection by day 26. Peritoneal cells from infected 5-LO^−/−^ mice also showed an enhanced production of PGE_2_ (Figure 1(b)) but the pattern was different than observed for WT cells and did not suggest shunting of arachidonic acid towards the cyclooxygenase pathway. PGE_2_ production by 5-LO^−/−^ cells was not elevated on day 5 and increased on day 12 (albeit to levels below WT) to a level that was maintained throughout infection. Notably, the peritoneal cells from both WT and 5-LO^−/−^ *T. cruzi*-infected mice appeared to produce more PGs than LTs, as evidenced by the fact that LTB_4_ levels (Figure 1(a)) were far lower than those of PGE_2_ (Figure 1(b)).

### 3.2. Control of Parasite Dissemination and Host Survival after Infection

As shown in Figure 1(c), infected 5-LO^−/−^ mice presented a delay in the appearance of blood-circulating parasites and very low parasite numbers at the second peak of parasitaemia compared to WT mice. In addition, the number of intact parasite nests in heart muscle tissue was considerably lower in 5-LO^−/−^ mice (Figure 1(d)). Furthermore, about 17% of the 5-LO^−/−^ mice died on postinoculation days 16–19, whereas WT mice did not begin to die until day 19 (Figure 1(e)). In the acute phase of infection, only 30% of WT mice were capable of surviving the infection. In contrast, 82.3% of 5-LO^−/−^ mice controlled parasite efficiently and survived the acute phase of infection (Figure 1(e)).

### 3.3. Inflammatory Infiltrate and Tissue Parasitism during the Acute Phase of* T. cruzi* Infection

**Figure 2:**
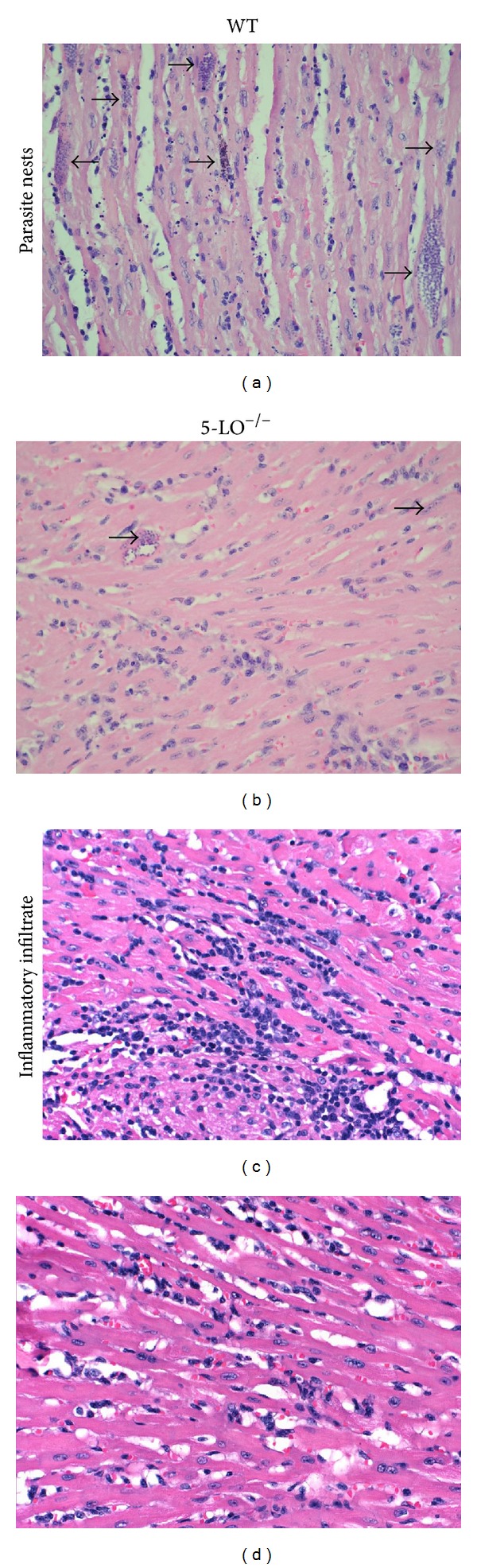
Histopathological analysis of heart tissue parasitism and inflammatory infiltrate in* T. cruzi*-infected WT and 5-LO^−/−^ mice (*n* = 8/group) on postinoculation day 16 (staining with H&E; magnification, ×200): (a) section from a WT mouse, showing numerous large amastigote nests, (b) section from a 5-LO^−/−^ mouse, showing fewer, smaller nests, (c) section from a WT mouse, showing intense mononuclear cell infiltration, (d) section from a 5-LO^−/−^ mouse, showing less infiltration. Photomicrographs are from one experiment representative of three independent experiments.

Analysis of the histological samples of heart muscle tissue collected after 16 days after inoculation revealed that WT mice presented more intact parasite nests and more amastigote forms within those nests (Figure 2(a)) than did 5-LO^−/−^ mice (Figure 2(b)). In addition, there were greater inflammatory mononuclear cell infiltrates in WT mice (Figure 2(c)) than in 5-LO^−/−^ mice (Figure 2(d)).

### 3.4. Cytokine Production by Spleen Cells

**Figure 3:**
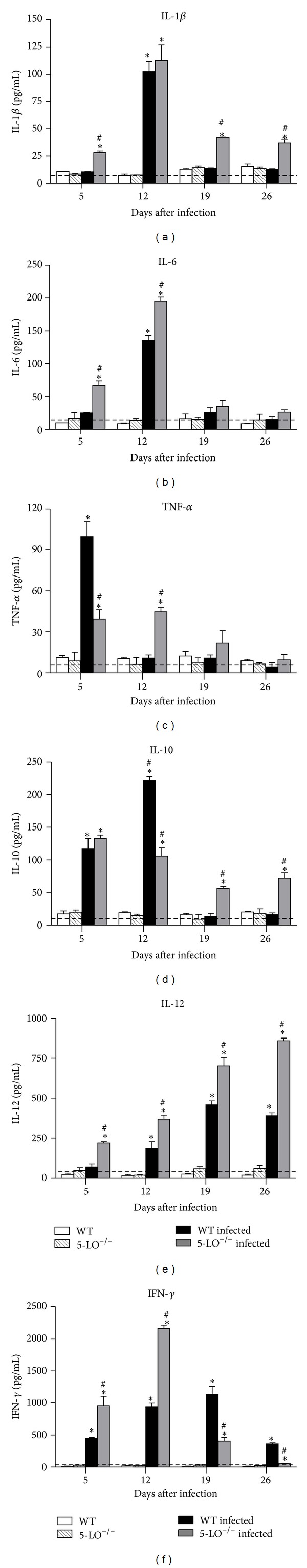
Cytokine production by spleen cells from* T. cruzi*-infected mice. Spleen cells from control, infected WT and infected 5-LO^−/−^ mice (*n* = 10/group) cultured in medium alone. Data are from one of three independent experiments. Kruskal-Wallis test (*P < 0.01 versus uninfected group; ^#^ *P* < 0.05 versus infected WT mice).

In culture, the spleen cells of infected mice spontaneously produced IL-1*β*, IL-6, TNF-*α*, IL-10, IL-12, and IFN-*γ* (Figure 3). It is notable that the production of most of these cytokines was greater during the first two weeks of infection, peaking on day 12 after inoculation, correlating with parasitemia (Figure 1(c)). In general, the production of IL-6, IL-12, and IFN-*γ* was greater in spleen cells of infected 5-LO^−/−^ than in WT cells. On day 5, the production of IL-1*β*, IL-6, IL-12, and IFN-*γ* was higher from 5-LO^−/−^ cells than from WT cells but TNF-*α* production was lower. On day 12, IL-1*β* levels were comparably high in cell cultures from both mouse strains, while spleen cells from infected 5-LO^−/−^ showed significantly greater production of IL-6, IL-12, IFN-*γ*, and TNF-*α* compared to the infected WT cells. The production of IL-10 showed the opposite trend (Figure 3(d)). In the late phase of infection (on days 19 and 26), 5-LO^−/−^ spleen cells, in contrast to what was observed for WT spleen cells, showed a sustained elevation in the production of IL-1*β* (Figure 3(a)) and IL-10 (Figure 3(d)), higher levels of IL-12 (Figure 3(e)), and lower levels of IFN-*γ* (Figure 3(f)).

### 3.5. Cell Populations in the Spleen of Infected Mice

**Figure 4:**
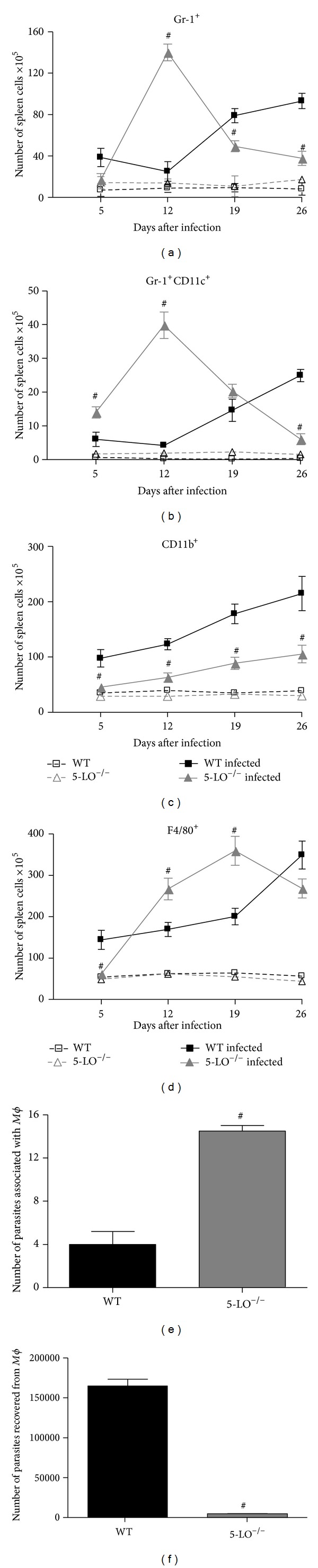
Quantitative and functional leukocyte responses to* T. cruzi* infection. (a) Gr1^+^ cell (neutrophils); (b) Gr1^+^CD11c^+^ cell (pDC cells); (c) CD11b^+^ cell (myeloid lineage cell marker, Mac-1); and (d) F4/80^+^ cell (macrophages) numbers in the spleen.

Infection of mice with* T. cruzi* led to the accumulation of Gr1^+^ cells (i.e., neutrophils), Gr1^+^/CD11c^+^cells (i.e., plasmacytoid dendritic cells), CD11b^+^cells (i.e., myeloid lineage cells), and F4/80^+^cells (i.e., macrophages) in their spleens when compared with uninfected control mice (Figures 4(a)–4(d)). Greater numbers of Gr1^+^, Gr1^+^/CD11c^+^, CD11b^+^, and F4/80^+^ cells were present in 5-LO^−/−^ than in WT spleens at day 12 after infection (Figures 4(a)–4(c)). Macrophage (F4/80^+^) numbers were higher in the spleens of 5-LO^−/−^ mice on day 19 after infection compared with WT mice as well (Figure 4(d)). In addition, on day 5 after infection, plasmacytoid dendritic cell (Gr1^+^/CD11c^+^) counts were higher in infected 5-LO^−/−^ mice, when compared with infected WT mice (Figure 4(b)). It is notable that myeloid lineage cells numbers were higher in the spleens of infected WT animals than the spleens of 5-LO^−/−^ mice (Figure 4(c)).

### 3.6. Peritoneal Macrophage Infection

In the infected 5-LO^−/−^ mice, PMs and IFN-*γ* both increased (Figures 4(d) and 3(f)). The* in vitro* infection of peritoneal LPS-plus IFN-*γ*-activated-macrophages from WT and 5-LO^−/−^ mice showed differences in the association of macrophages with parasites (binding and internalization) and in their ability to kill intracellular parasites (Figures 4(e) and 4(f)). Compared with the activated PMs from WT mice, those from 5-LO^−/−^ mice presented a greater capacity to associate with the blood form of the parasite, as evidenced by the higher numbers of bound and internalized parasites (Figure 4(e)). Activated PMs from 5-LO^−/−^ mice were also more efficient at killing internalized parasites, as evidenced by the lower numbers of parasites recovered after* in vitro* infection as compared with WT PMs (Figure 4(f)).

### 3.7. Spleen B-Cell Counts and Serum Levels of Parasite-Specific Immunoglobulins

**Figure 5:**
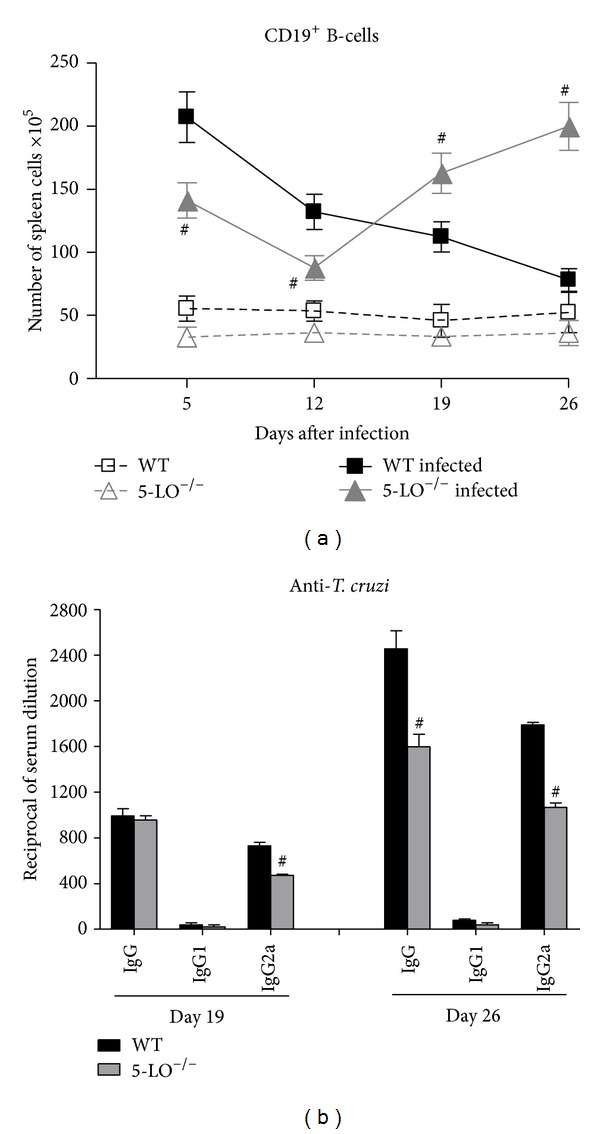
(a) Splenic CD19^+^ B-cell counts in control, infected WT and infected 5-LO^−/−^ mice (*n* = 10/group). Data are from one of three independent experiments. Student's *t*-test (^#^ *P* < 0.01 versus infected WT mice). (b)* T. cruzi*-specific serum antibody titers in infected WT and 5-LO^−/−^ mice (*n* = 10/group). Data are from one of two independent experiments. ^#^ *P* < 0.001 versus infected WT mice.

As shown in Figure 5(a), splenic CD19^+^ B-cell counts were greater in mice infected with* T. cruzi* than in control mice. On day 5 after inoculation, splenic CD19^+^ cell counts were elevated in infected WT mice and gradually returned to baseline values by the end of the acute phase of infection. In contrast, splenic CD19^+^ cell counts increased significantly less in infected 5-LO^−/−^ mice during the first two weeks of infection, gradually becoming significantly more elevated than WT mice in the later phase of infection.

Mice infected with* T. cruzi* produced detectable levels of parasite-specific IgG antibodies in a time-dependent manner during the later phase of infection (Figure 5(b)). The principal isotype produced during infection was IgG2a, and levels of IgG1 were low. Compared with WT mice, 5-LO^−/−^ mice presented lower levels of parasite-specific IgG on day 26, as well as of parasite-specific IgG2a on days 19 and 26 of infection.

### 3.8. T-Cell Phenotypes in the Spleens of Infected Mice

**Figure 6:**
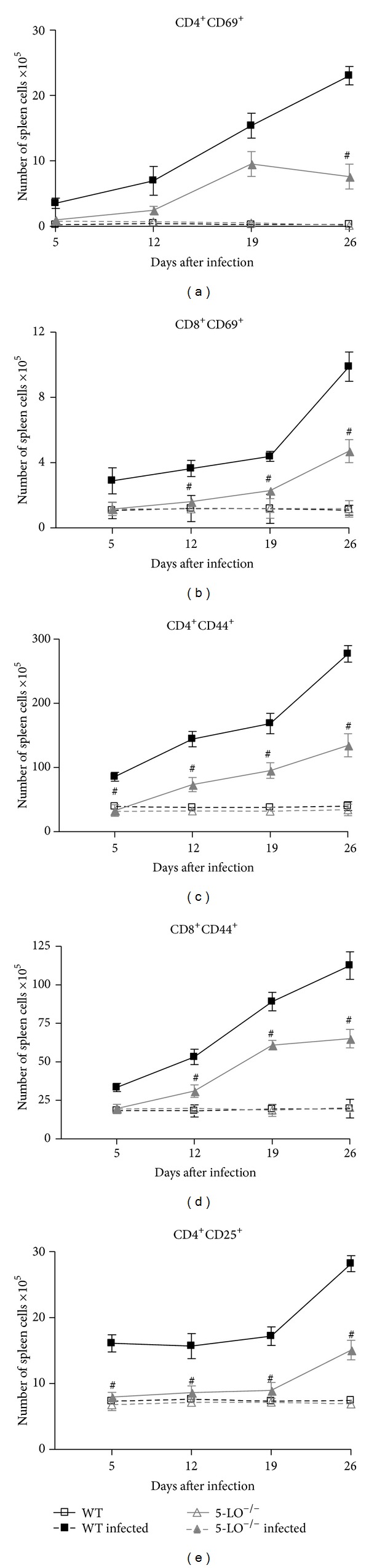
Splenic T-cell subpopulations in control, infected WT and infected 5-LO^−/−^ mice (*n* = 8/group). Data are from one of three independent experiments. Student's *t*-test (^#^ *P* < 0.01 versus infected WT mice).

As indicated in Figure 6, infected mice presented elevated CD4^+^ and CD8^+^ T-cell counts, the markers CD4^+^CD69^+^, CD8^+^CD69^+^, CD4^+^CD25^+^, CD4^+^CD44^+^, and CD8^+^CD44^+^, which indicate the presence of activated T-cells in the spleens of both WT and 5-LO^−/−^ infected mice. In infected mice, the majority of the splenic T-cell populations presented the full/late T-cell activation markers CD4^+^CD44^+^ and CD8^+^CD44^+^ (Figures 6(c) and 6(d)). On day 5 after inoculation, numbers of all of these T-cell phenotypes were elevated in infected WT mice, gradually increasing over the course of infection and peaking on day 26, the study endpoint (Figure 6). In contrast, infected 5-LO^−/−^ mice presented a delayed elevation in T-cell counts, and the increase of all of these activated T-cell phenotypes was less pronounced than observed in WT spleens. Infected 5-LO^−/−^ mice presented a significant increase in CD4^+^CD44^+^ and CD8^+^CD44^+^ counts on day 12, a marked increase in the CD4^+^CD69^+^ count on day 19, and a slight but significant increase in CD8^+^CD69^+^ and CD4^+^CD25^+^ counts only on day 26.

### 3.9. T-Cells Properties, Cytokine Production, and CD4^+^ Memory T Cells Expressing CD45RB^low^ and CD44^high^CD62L^low^in the Spleen

**Figure 7:**
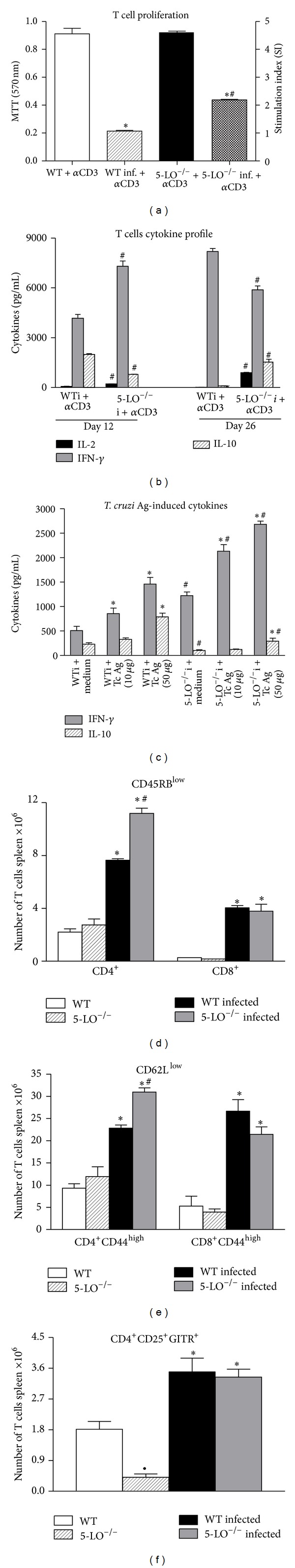
Splenic T-cell properties, effector/memory T cells, and regulatory T cells in control, infected WT and infected 5-LO^−/−^ mice (*n* = 8/group): (a) proliferation; (b) cytokine production profile after anti-CD3 stimulation; and (c) Th1/Th2 cytokine recall response to soluble* T. cruzi* antigen. *P < 0.001 versus uninfected group; ^#^ *P* < 0.001 versus infected WT mice. (d) CD4^+^CD45RB^low^ and CD8^+^CD45RB^low^; (e) CD4^+^CD44^high^CD62L^low^ and CD8^+^CD44^high^CD62L^low^; and (f) CD4^+^CD25^+^GITR^+^. Stimulation index (SI) was generated by the ratio between the OD (570 nm) obtained in noninfected/infected cells. Data are from one of two independent experiments. Student's *t*-test (^#^ *P* < 0.01 versus infected WT mice).

As shown in Figure 7(a), spleen cells from control mice proliferated after stimulation with anti-CD3*ε*, as expected, whereas spleen cells collected from infected WT mice on day 12 after inoculation and stimulated with anti-CD3*ε* presented a dramatic reduction in proliferation. However, spleen cells from infected 5-LO^−/−^ mice on day 12 and stimulated with anti-CD3*ε* presented a partially restored proliferative capacity.

Spleen cells were collected from infected mice on days 12 and 26 after inoculation, after which they were stimulated with anti-CD3*ε*. The supernatants were tested for the presence of IFN-*γ* and IL-10, the most abundant of the cytokines secreted spontaneously by spleen cells from infected mice that we measured (Figure 3), as well as for IL-2. Spleen cells collected from infected WT mice on day 12 and stimulated with anti-CD3*ε* produced significant amounts of the type 1 cytokine (Th1) IFN-*γ*, and the type 2 cytokine (Th2) IL-10, as well as very low levels of IL-2 (Figure 7(b)). In contrast, anti-CD3*ε*-stimulated spleen cells collected from infected 5-LO^−/−^ mice on day 12 presented a bias to produce predominantly, and in greater quantities, Th1 cytokines, producing lower quantities of Th2 cytokines and greater quantities of IL-2.

On day 26 after inoculation, infected WT mouse spleen cells stimulated with anti-CD3*ε* exhibited a bias to produce only the Th1 cytokine IFN-*γ* in quite high amounts (Figure 7(b)). However, infected 5-LO^−/−^ mouse spleen cells receiving the same treatment presented no alterations in the cytokine production profile, a sustained capacity to produce detectable levels of IL-2, and high (and predominant) levels of Th1 cytokines, as well as low levels of Th2 cytokines, on day 12.

Since anti-CD3*ε* promotes a polyclonal T-cell stimulation, we investigated whether IFN-*γ* and IL-10 were produced by primed* T. cruzi*-specific cells. We tested the recall responses of spleen cells collected on day 12 after inoculation and cultured with soluble* T. cruzi* antigens.* T. cruzi*-specific cells from infected WT mice cultured with nominal antigens produced IFN-*γ* and IL-10, whereas* T. cruzi*-specific cells from infected 5-LO^−/−^ mice produced a recall response, characterized by high levels of IFN-*γ* and lower levels of IL-10, in the same culture.

These results regarding the recall response suggested the presence of functional parasite-specific effector/memory T cells. Therefore, we sought to study the populations of effector/memory T cells. As shown in Figure 7, infected mice exhibited increased numbers of splenic effector/memory T cells in CD4^+^ and CD8^+^ subsets, including CD4^+^CD45RB^low^, CD4^+^CD44^high^CD62L^low^, CD8^+^CD45RB^low^, and CD8^+^CD44^high^CD62L^low^, on day 26 after inoculation. Interestingly, the numbers of effector/memory T cells CD4^+^CD45RB^low^ and CD4^+^CD44^high^CD62L^low^ were higher in infected 5-LO^−/−^ mice than in infected WT mice. However, we found no differences between infected 5-LO^−/−^ mice and infected WT mice in terms of the numbers of CD8^+^ effector/memory T cells.

We questioned whether the kinetic differences between infected 5-LO^−/−^ mice and infected WT mice in terms of the increase in CD4^+^CD25^+^ T cell numbers (Figure 6(e)) were related to the involvement of regulatory T cells in this model.  Figure 7(f) shows that* T. cruzi*-infected mice exhibited increased numbers of CD4^+^CD25^+^GITR^+^regulatory T cells. However infected WT mice and 5-LO^−/−^ mice exhibited similar numbers of splenic CD4^+^CD25^+^GITR^+^ regulatory T cells. Unexpectedly, the numbers of CD4^+^CD25^+^GITR^+^ in the spleen were lower in uninfected 5-LO^−/−^ mice than in uninfected WT mice.

### 3.10. Levels of LTC_4_, Serum Nitrite Levels, and Protein Extravasation into the Peritoneal Cavity during the Acute Phase of* T. cruzi* Infection

**Figure 8:**
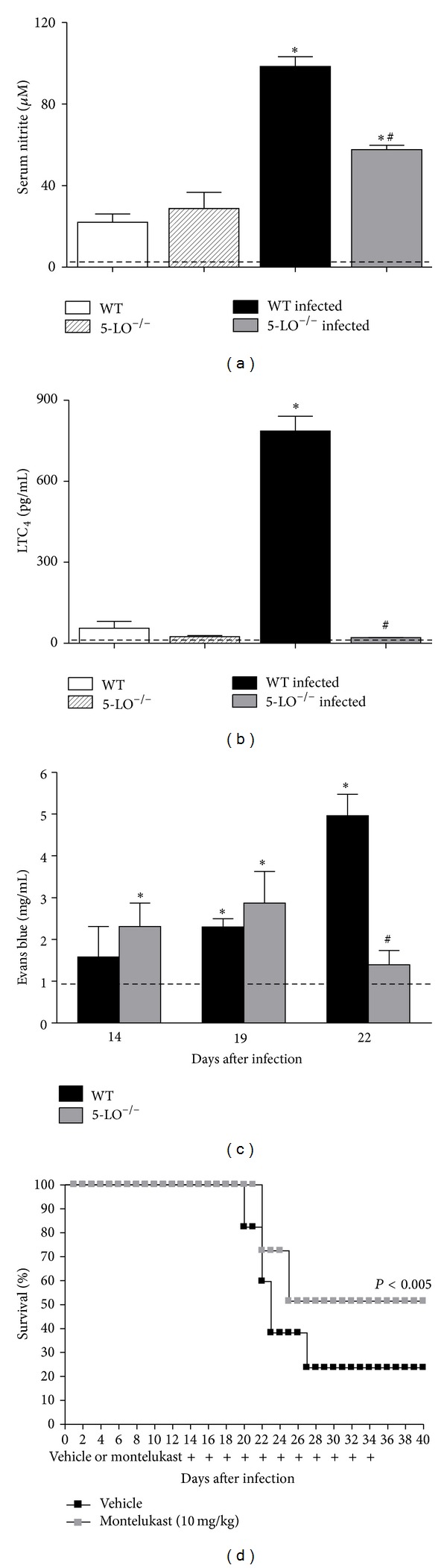
Levels of vasoactive/vasomotor mediators and relevance of cys-LTs in mice acutely infected with* T. cruzi *(*n* = 10/group): (a) total serum nitrite (nitrite/nitrate) levels and (b) LTC_4_ production by peritoneal cells upon stimulation with calcium ionophore. Data are from one of three independent experiments. ^#^ *P* < 0.001 versus infected WT mice. (c) Protein extravasation in the peritoneal cavity (colorimetric assay of extravascular dye leakage). Dotted line indicates the mean Evans blue D.O. found in control mice. Data are from one experiment representative of three separate experiments. ^#^ *P* < 0.05 versus infected WT mice. (d) Effect of cys-LT receptor antagonist on survival in* T. cruzi*-infected mice: (black squares) vehicle-treated infected WT mice and (gray squares) infected WT mice treated with montelukast (10 mg/kg) from postinoculation day 14 to postinoculation day 34 and monitored for 26 days. Data are from one experiment representative of two separate experiments. Wilcoxon signed-rank test ^#^ *P* < 0.005 versus vehicle-treated infected WT mice.

The cys-LTs mediate detrimental vascular effects in systemic infections such as sepsis [23]. Vasoactive mediators are produced and may also be involved in the mortality of animals during the acute phase of* T. cruzi* infection [35]. Thus, we next examined the levels of some vasoactive mediators such as NO metabolites, LTC_4_, and measured the protein leak (as a marker of vascular permeability) at a time point when infected WT mice have a high mortality while infected 5-LO^−/−^ mice do not. As illustrated in Figure 8, the capacity to produce LTC_4_ was markedly upregulated in the peritoneal cells of infected WT mice on day 22 after inoculation, although, as expected, those of infected 5-LO^−/−^ mice produced no detectable levels of LTC_4_. At this time point, infected 5-LO^−/−^ mice presented significantly lower serum nitrite levels than did infected WT mice. In addition, the peritoneal cavity protein extravasation assays (Figure 8(c)) indicated that on days 14 and 19, the degree of protein leakage was similar between the two groups of infected animals. Although the 5-LO^−/−^ mice presented a tendency toward greater protein leakage than did the WT mice, the difference was not significant. However, on day 22, protein leakage in the peritoneal cavity was considerably greater in the infected WT mice than in the infected 5-LO^−/−^ mice.

### 3.11. Treatment of Infected Mice with cys-LTs Receptor Antagonist and Mortality

To determine whether cysLTs are involved in the mortality of* T. cruzi*-infected mice, WT mice were subjected to infection with* T. cruzi* and treated with the cysLT receptor antagonist montelukast from day 14 to day 34 after inoculation. On day 40 after inoculation, when mortality among the vehicle-treated control WT mice was 82%, a moderately significant degree of protection (reduction to 48% mortality) was achieved after treatment with montelukast (Figure 8(d)).

## 4. Discussion

Leukotrienes, products of the 5-LO pathway of arachidonic acid metabolism, are potent immunomodulatory lipids that are increasingly recognized to regulate innate and adaptive immune responses to parasitic infections [36]. Despite the relevance of LTs in* T. cruzi* killing by macrophages* in vitro* as well as in controlling blood parasite numbers* in vivo* [26, 37, 38], the oxidative balance in 5-LO^−/−^ *T. cruzi* infected mice has been related to be 5-LO-pathway independent [39]. Different results for NO and cytokines productions were observed during the acute phase of* T. cruzi* Y strain infection of 5-LO^−/−^ mice and it has been related to resistance [40] or susceptibility [41]. The localized presence of TCD8^+^ or TCD4^+^ in the heart was demonstrated in* T. cruzi *5-LO^−/−^ infected mice [40], but other cell types and/or eicosanoid mediators were not investigated for a more complete immune response analysis. The novel finds in this study focus on the* in vivo* role of 5-LO metabolites in cells related to innate/adaptive immune responses, resistance, and mortality during the acute phase of* T. cruzi* murine infection. It is important to note that analysis performed after day sixteen after infection was done using infected mice that had survived up to that time point. Furthermore, we describe the results of 5-LO^−/−^ mice infection, using* T. cruzi* Colombian strain, contributing to a better understanding of the immune response and pathology of the Chagas' disease.

Our data show that peritoneal cells from infected WT mice develop an enhanced capacity to produce LTB_4_, LTC_4_, and PGE_2_ compared with cells obtained from uninfected mice, implicating LTs and PGs in the host response to* T. cruzi* parasitic infection. Compared with WT, 5-LO^−/−^ mice developed significantly reduced parasitemia, lower tissue parasitism, and less inflammatory cell infiltrates, as well as a significant improvement in survival. Our curve of parasitemia showed a different profile compared with two previous publications [40, 41]. However, these publications are also different between them, what could be due to different trypomastigotes that were used to infect the experimental groups of mice, since one work used cell culture-derived trypomastigotes [40] and other used mice-derived trypomastigotes [41]. Moreover, both works used the Y strain of* T. cruzi*, while we used the Colombian strain.

These scenarios suggest that LTs deficiency renders mice more resistant to* T. cruzi* infection and conversely that 5-LO products confer susceptibility to* T. cruzi* virulence. The production of proinflammatory cytokines IL-1*β*, IL-6, IL-12, TNF-*α*, and IFN-*γ* and the presence of parasite-specific T cells generating predominantly IFN-*γ* and low levels of IL-10 were associated with an increased efficiency of 5-LO^−/−^ mice to control the infection within the blood and tissue compartments. In fact, in the later phases of infection, parasitemia and tissue parasitism were significantly reduced in 5-LO^−/−^ mice and it is in accordance with previous studies showing that cytokines such as IL-1*β*, IL-6, IL-12, TNF-*α*, and IFN-*γ* play a relevant role in host killing mechanisms against* T. cruzi* [8].

Consistent with our data, there is evidence that LTs induce TNF-*α* [38] and PGE_2_ [42] release. In some models, it has been observed that drug-induced or genetic LTs deficiency increased PGE_2_ levels [43]. We observed that 5-LO^−/−^ mice presented a sustained potential to produce PGE_2_ in the late phase of infection. In support of our findings that LTB_4_ induced IL-6, healthy patients subjected to inhalation of swine house dust and treated with 5-LO inhibitor showed elevated IL-6 serum levels [44]. In addition, LTB_4_ might induce IL-1*β* production [45]; although it is not seen as a unique inducer, we can presume that, in 5-LO^−/−^ mice, IL-1*β* and IL-6 were induced by* T. cruzi* PAMPs (Pathogen Associated Molecular Patterns), as previously described [46].

In some murine models of fungal or bacterial infection, it was suggested that the pharmacological impairment of LT biosynthesis hindered the production of the Th1 cytokines IL-12 and IFN-*γ* [20, 21]. However, in our model, 5-LO^−/−^ *T. cruzi* infected mice exhibited an increased capacity to produce IL-12 and IFN-*γ*. A similar result was obtained in other infection models using 5-LO^−/−^ mice [22, 29, 30], and this capacity was found to be essential to achieving protective immunity against pathogens in these mice [22, 47]. Previous studies have demonstrated that the quality and quantity of inflammatory mediators such as IL-12, IFN-*γ*, and IL-10 released during the first two weeks of infection are critical to driving the generation of parasite-specific effector T cells [48] and we suggest that early IL-12 and IFN-*γ* production during infection was regulated by LTs. It is probable that splenic Gr-1^+^CD11c^+^ plasmacytoid dendritic cells, Gr-1^+^ neutrophils, and F4/80^+^ macrophages are sources of IL-12 and IFN-*γ*, since the numbers of these cells were found to be significantly higher in 5-LO^−/−^ infected mice than in WT infected mice. These cell types have also been found to be increased by* T. cruzi* infection in other models [10, 49], and they are relevant source of IL-12 and IFN-*γ* in the setting of protozoal infection [50, 51]. Furthermore, IL-12-producing CD11c^+^ cells were found to be elevated in 5-LO^−/−^ model of* M. tuberculosis* infection [22].

There is evidence that LTs contribute to the process of T-cell activation/migration in different models [52, 53]. Our results demonstrated the importance of 5-LO products in T-cell activation during* T. cruzi* infection. Although a reduction in the numbers of activated splenic T cells was achieved in the infected 5-LO^−/−^ mice, the T cells from these animals, as opposed to those from WT mice, presented a partially recovered capacity to proliferate after anti-CD3*ε* stimulation and also to produce IL-2 after anti-CD3*ε* stimulation or in the presence of* T. cruzi* soluble antigens. We are in accordance with previous studies showing that LTs may also inhibit T cell proliferation and IL-2 production [54] and that susceptibility to* T. cruzi* infection is associated with elevated numbers of polyclonal activated T cells in the spleen [55] or with splenic T cell unresponsiveness to mitogens and inability to secrete IL-2 [56]. Furthermore, the resistance to* T. cruzi* infection of 5-LO^−/−^ mice, in contrast to the susceptibility of WT mice, correlated with elevated numbers of splenic effector/memory T cells, including CD4^+^CD45RB^low^ and CD4^+^CD44^high^  CD62L^low^, at the end of the acute phase of infection. Elevated numbers of T cells expressing these phenotypes have been associated with IFN-*γ* production and resistance to* T. cruzi* infection [11].

The correlation between the resistance to infection and the Th1 bias of CD4^+^ T cells has been identified in 5-LO^−/−^ mice infected with other pathogens, including* M. tuberculosis *[22] and* T. gondii *[57]. The bias towards IFN-*γ* production by T cells from 5-LO^−/−^ mice was also observed following infections with typical Th2-inducing pathogens such as* S. mansoni* [29] and* S. venezuelensis *[30] leading these animals to become more resistant and susceptible to infection, respectively. The CD4^+^CD25^+^ T cells number abnormality found in* T. cruzi* 5-LO^−/−^ infected mice led us to further investigate these CD4^+^CD25^+^ regulatory T cells. We found that WT and 5-LO^−/−^ infected mice presented similar numbers of splenic CD4^+^CD25^+^GITR^+^ regulatory T cells. This does not completely rule out the involvement of CD4^+^CD25^+^ regulatory T cells in the present model. In fact, it was recently demonstrated that CD4^+^CD25^+^ regulatory T cells play a limited role during the acute and chronic phases of* T. cruzi* infection [58].

Phagocytes have long been known to play an important role in the* T. cruzi* killing process [59]. It is also known that IFN-*γ* is one of the major mediators conferring resistance to* T. cruzi* [60]. Macrophage (F4/80^+^) numbers and IFN-*γ* were found to be increased in* T. cruzi* 5-LO^−/−^ infected mice.* In vitro *infection assays revealed that activated PMs from 5-LO^−/−^ mice were strongly associated with more efficient parasite killing than WT macrophages. These results corroborate our* in vivo* findings that 5-LO^−/−^ mice are more efficient at controlling parasitemia, but contrasts with previous* in vitro* findings showing LTs foster intracellular parasite killing [26, 38]. However, it is suggested in 5-LO^−/−^ mice that an oxidative stress occurs by a leukotriene-independent pathway, since an increase in erythrocyte oxidative stress was observed in these animals [39]. Differences in experimental design between our study and previously published investigations warrant discussion. Some differences in results may be explained in part by the macrophage activation and/or responsiveness to LTs. We used classic activated macrophages (M1)* in vitro* [61], whereas previous studies showing enhanced pathogen-killing properties of LT-stimulated macrophages employed resident peritoneal macrophages [26, 37], thioglycollate-elicited macrophages [28, 38], or alveolar macrophages [18]. The functional differences among these different types of macrophages are remarkable and have been consistently described [62, 63]. The increased capacity of macrophages from 5-LO^−/−^ mice to kill intracellular pathogens was previously described for* M. tuberculosis* [22]. These findings underscore the relevance of IFN-*γ* and the killing activity of macrophages in 5-LO^−/−^ mice resistance to* T. cruzi* infection, although our data shed no light on whether LXA_4_ is involved in the parasite resistance of mice, as has been described for* M. tuberculosis* infection [22].

Analysis of B cells indicated that* T. cruzi* 5-LO^−/−^ infected mice developed smaller increases in the numbers of splenic CD19^+^ B cells during the first two weeks than did WT mice, but this was followed by increased numbers of splenic CD19^+^ B cells in the following weeks. The elevated numbers of CD19^+^ B cells found in 5-LO^−/−^ mice in the later phase of infection might indicate an accumulation of undifferentiated B cells in the splenic compartment. This hypothesis is supported by previous studies showing that antibody-secreting B cells lose their CD19 marker [64]. Previous* in vitro* findings showed that LTs are important in activating B cells in human and mouse models [65, 66] and suggest that this could be also relevant* in vivo*. The alteration in B cell activation observed in* T. cruzi* 5-LO^−/−^ infected mice could explain the lower serum levels of parasite-specific IgG and IgG2a at the end of the acute phase of infection. It was previously described that LT deficiency altered specific-immunoglobulin class switching to specific pathogens* in vivo* [29, 30]. Indeed, LTs may affect parasite-specific antibody levels during infection and parasite-specific IgG and IgG2a might not be involved in 5-LO^−/−^ mouse resistance to* T. cruzi* infection. This reinforces the previous observation that host resistance during the acute phase of* T. cruzi* infection can be achieved in the absence of B cells [67].

Animal mortality during the acute phase of* T. cruzi* infection has been associated with multiple factors, including parasite strain virulence [68], anemia [69], increased levels of TNF-*α*, and T-cell hyperactivity [70]. In fact,* T. cruzi*-infected mice have been shown to be extremely sensitive to sepsis-like inducers and to die with evidence of a shock syndrome [69]. We demonstrated that* T. cruzi*-infected WT mice presented, at the acute phase, an upregulated production of LTB_4_ and LTC_4_, as well as high serum nitrite levels. In addition, the analysis of protein extravasation in the peritoneal cavity revealed that infected WT mice exhibited stronger protein leakage as compared with 5-LO^−/−^ mice. Previous studies showed that LTs induce NO production in macrophages and endothelial cells [38], and high levels of NO production have been associated with increased mortality in* T. cruzi*-infected mice [71]. LTs play a critical role in vascular events and mortality of mice subjected to the cecal ligation and puncture model of sepsis [23]. We observed that* T. cruzi-*infected WT mice treated with montelukast presented a significant reduction in mortality, providing evidence that cys-LTs are involved in vascular events associated with mortality of animals during the acute phase of* T. cruzi* infection. It is notable that montelukast treatment was less effective in increasing survival in contrast to what is observed in* T. cruzi-*infected 5-LO^−/−^ mice. The deaths of some montelukast-treated animals might be attributable to the presence of other 5-LO products, such as LTB_4_, and its indirect effect of inducing vasoactive mediators such as NO [72] and thromboxane [73]. However, unlike WT, 5-LO^−/−^ infected mice sustain the production of TNF-*α* and IL-6. This finding is not surprising, since these cytokines have been shown to have vasoactive properties [35].

One reasonable mechanism to explain WT mice mortality during the acute phase of* T. cruzi* infection involves their capacity to produce LTs, which lead to the extreme bias of spleen cells and T-cells that secrete high levels of Th1 cytokines, such as IFN-*γ*, and very low levels of IL-10. In addition, downregulated production of PGE_2_ might be relevant, since PGE_2_ has been shown to induce IL-10 production [74], inhibit the production of IFN-*γ* during* T. cruzi* infection, and promote the increased production of NO [75]. The advantage in survival of* T. cruzi*-5-LO^−/−^ infected mice represents a complex contribution of various effects on host defense, including their capacity to efficiently control the parasites and produce detectable levels of IL-10, the increase in production of PGE_2_, and lesser amounts of NO, as well as their inability to produce cys-LTs.

## 5. Conclusions

Our findings demonstrated that 5-LO deficiency altered eicosanoids and cytokines production during* T. cruzi *infection and favored the generation/maintenance of protective immune responses. Also, they provided evidence that 5-LO-derived lipid mediators have a negative effect on host survival during the acute phase of infection.
